# Intraperitoneal Gemcitabine Chemotherapy Treatment for Patients with Resected Pancreatic Cancer: Rationale and Report of Early Data

**DOI:** 10.1155/2011/161862

**Published:** 2011-12-12

**Authors:** Paul H. Sugarbaker, O. Anthony Stuart, Lana Bijelic

**Affiliations:** Washington Cancer Institute, 106 Irving Street, NW, Suite 3900, Washington, DC 20010, USA

## Abstract

Currently, the surgical management of pancreas cancer is recognized around the world as inadequate. Despite a potentially curative R0 resection, long-term survival is rare. There is a strong rationale for the use of chemotherapy in the operating room to reduce local-regional of recurrent/progressive disease. Gemcitabine monotherapy administered by an intraperitoneal route in the operating room with hyperthermia and then for long-term treatment postoperatively has a pharmacologic basis in that the exposure of peritoneal surfaces to intraperitoneal gemcitabine is approximately 200–500 times the exposure that occurs within the plasma. A standardized treatment with intraoperative and long-term chemotherapy that is well tolerated would greatly facilitate further improvements in pancreas cancer treatment and may lead the way to an evolution of more successful treatment strategies of this dread disease. The aim of this paper is to present the early data on a protocol in progress in patients with resected pancreatic cancer.

## 1. Introduction

Pancreatic cancer is the fourth leading cause of cancer-related deaths in the United States of America with an estimate of 37,660 deaths in 2011 [1]. Surgery represents the only definitive treatment option, and complete tumor resection is associated with better disease-free and overall patient survival. Advances in surgical technique, anesthesia, and perioperative care in the last two decades have led to a substantial decrease in perioperative mortality and morbidity especially in large-volume centers. Unfortunately, only 10–20% of patients diagnosed with pancreatic cancer are able to undergo potentially curative surgery [2]. Furthermore, long-term 5- or 10-year survival is rare, even after potentially curative R0 resection. Recently, Cleary reported 18 of 123 (15%) 5-year survival; 4 of these 18 patients died of disease after 5 years [3]. After curative resection, disease recurrence has been documented in the local and regional area (50%), on peritoneal surfaces (40–60%) and within the liver as hepatic metastases (50–60%) [4].

### 1.1. Rationale for Intraperitoneal Gemcitabine

The mechanisms of failure after pancreaticoduodenectomy are unclear. One possible explanation for the large number of local and regional failures is tumor dissemination and implantation within the resection site during surgery. Conceptually, this forms the basis for administration of perioperative and long-term intraperitoneal chemotherapy. The major advantage of intraperitoneal chemotherapy is the high drug level that can be achieved locally with low systemic exposure [5]. A systematic review of randomized control trials has established the role of adjuvant perioperative intraperitoneal chemotherapy in high-risk gastric cancer patients after potentially curative resection [6]. Also, long-term intraperitoneal chemotherapy has established efficacy in ovarian cancer [7–9]. Success of systemic chemotherapy in controlling local disease has a weaker rationale and has never been confirmed in randomized trials. The pharmacokinetics of gemcitabine makes it an excellent drug for intraperitoneal use. With evidence mounting for use of intraperitoneal chemotherapy after resection in ovarian and gastric cancer, a rationale for the use of intraperitoneal chemotherapy after curative resection in pancreatic cancer should be explored with a formal protocol.

## 2. Washington Cancer Institute Phase II Study of Adjuvant Intraperitoneal Gemcitabine for Resectable Pancreatic Adenocarcinoma: Methods and Early Results

In an Institutional Review Board-approved protocol (MHRI-GU-2009-455) we have initiated a local-regional treatment using gemcitabine monotherapy. After enrollment and informed consent, a standard pancreatic resection is performed, and, if necessary, there is pathologic confirmation of primary pancreatic adenocarcinoma. Patients with cancer of the head of the pancreas and tail of the pancreas who have a complete visible resection of disease are eligible.

**Figure 1:**
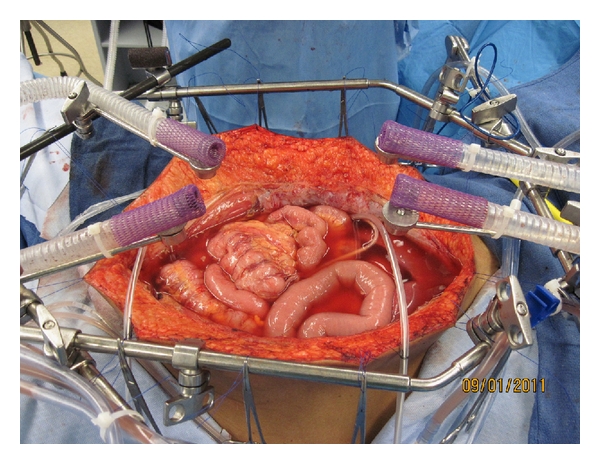
Hyperthermic intraperitoneal chemotherapy for treatment of abdominal and pelvic surfaces following pancreaticoduodenectomy. To administer hyperthermic intraperitoneal chemotherapy there is one inflow catheter and four drainage catheters. The chemotherapy solution is maintained at approximately 43°C at the inflow catheter and 41°C throughout the whole abdomen. Four smoke evacuators are placed around the periphery of the open abdomen in order to create a “vapor barrier” above the chemotherapy solution. The surgeon's double-gloved hand is used to maintain a uniform distribution of the heat and chemotherapy solution.

Following cancer resection gemcitabine at 1000 mg/m^2^ is instilled into the peritoneal cavity in a chemotherapy solution containing 1.5% dextrose peritoneal dialysis solution. The volume of peritoneal dialysis solution is 1.5 L/m^2^. There is a single inflow catheter that is placed in the anatomic site from which the pancreatic cancer was removed. Four outflow drains are positioned in the right upper quadrant, left upper quadrant, and two within the pelvis. A heater circulator (Belmont, Billerica, Mass) was utilized to maintain the chemotherapy solution at 43°C at the inflow and 41°C within the whole abdomen. The treatment lasts for 1 hour and there is an open technique used with a vapor barrier that allows continuous manipulation of the abdominal and pelvic contents by the surgeon and uniform distribution of the heated chemotherapy solution (Figure 1).

Prior to closing the abdominal incision an intraperitoneal port (Port-A-Cath, Smiths Medical MD, Inc., St. Paul, Minn) is positioned. The port is accessed with a noncoring right angle needle (Port-A-Cath Gripper Plus, Deltec, Inc., St. Paul, Minn) to temporarily maintain proper position for use in 4 to 6 weeks [10]. When the patient has fully recovered from surgery and the sutures have been removed from the skin incision, the adjuvant intraperitoneal gemcitabine is begun. There are six cycles, each of which is 4 weeks in length. Gemcitabine at 1,000 mg/m^2^ is given by intraperitoneal administration on days 1, 8, and 15 of the 4-week cycle.

Results to date show that the hyperthermic intraperitoneal gemcitabine and the long-term intraperitoneal gemcitabine are well tolerated. No grade III or IV toxicities were observed. To date, seven patients have been treated with hyperthermic gemcitabine as part of the pancreaticoduodenal resection, and the accrual process is ongoing.

**Figure 2:**
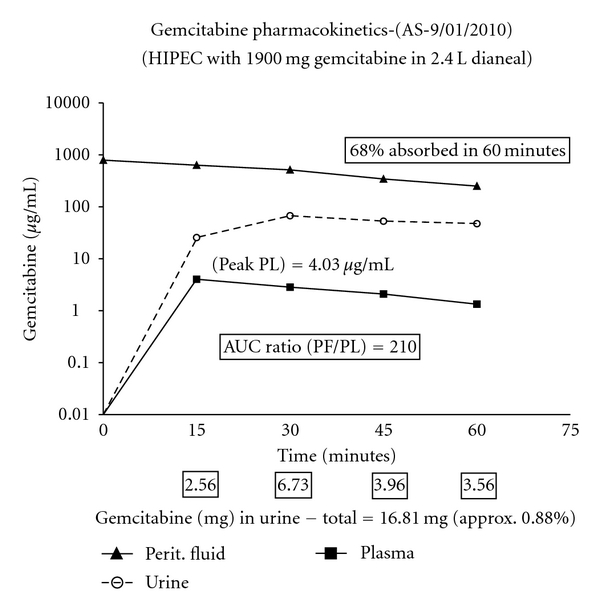
Pharmacology of intraoperative intraperitoneal gemcitabine in a patient with resected pancreas cancer. The drug was used at 1,000 mg/m^2^ in 3 liters of 1.5% dextrose peritoneal dialysis solution administered intraperitoneally. The area under the curve ratio of concentration × time intraperitoneal to intravenous was 210. Sixty-eight percent of the drug was cleared from the peritoneal cavity in 60 minutes. Data were taken from the study of a single patient but are similar to those in four other patients. The patient has completed the long-term intraperitoneal gemcitabine without incident.

As part of this phase I/II single institution study, a pharmacokinetic analysis of hyperthermic intraperitoneal gemcitabine is being performed. There is a standard dose of 1,000 mg/m^2^ of gemcitabine in a standard volume of 1.5% dextrose peritoneal dialysis solution (1.5 L/m^2^). Peritoneal fluid, plasma, and urine samples are obtained at 15-minute intervals throughout the 60 minutes of hyperthermic intraperitoneal chemotherapy. The results as seen in a single patient are presented in Figure 2. Similar data has been obtained in 4 additional patients. The area under the curve ratio of concentration times time of intraperitoneal to intravenous gemcitabine was 210. To date, no data regarding gemcitabine within pancreatic tissues is available.

Six months of normothermic intraperitoneal gemcitabine using an intraperitoneal port was selected as an analogy to the current recommendation for use of intravenous gemcitabine. Not only the schedule but also the doses of intraperitoneal chemotherapy are the same as currently recommended for systemic treatment. The route of administration in the current study is intraperitoneal rather than intravenous.

## 3. Discussion: Summary of Randomized Control Trials of Adjuvant Therapy for Pancreatic Cancer

Realizing that the chances are small of surgical resection alone being curative, there have been many studies analyzing the benefits of adjuvant therapy in pancreatic cancer. In 1985 the Gastrointestinal Study Group (GITSG) conducted a 2-arm study trial randomizing patients into 5-fluorouracil- (5-FU-) based chemoradiation versus observation [11]. The mean survival in the chemoradiation arm was 20 months compared to 11 months in the observation arm. The 5-year survival was 18% and 8%, respectively. The trial was able to recruit only 43 patients in 11 years and had to be prematurely closed due to slow accrual and significant benefit favoring adjuvant chemoradiation.

The European Organization for Research and Treatment of Cancer (EORTC) trial was an adequately powered study designed to validate the result of the smaller GITSG trial [12]. Adjuvant therapy was similar except that the GITSG study used maintenance chemotherapy while the EORTC trial did not. In the EORTC trial, 218 patients with pancreatic and ampullary cancer were recruited. Randomization was to the observation group or radiotherapy with split-course radiotherapy (40 Gy) and concurrent 5-FU as a continuous infusion. After a median followup of 11.7 years, there was no difference in overall survival between the 2 arms. The limitations of this study were the lack of maintenance chemotherapy and a questionable statistical design that limited its ability to detect a small benefit for adjuvant chemoradiation.

The European Study Group for Pancreatic Cancer (ESPAC) conducted a trial between 1994 and 2000 (ESPAC-1) [13]. In the 2 × 2 factorial design, 145 patients were randomized to the chemoradiotherapy arm, and 144 were randomly assigned to no chemoradiotherapy. Radiation was administered as a split course (total 50 Gy), concurrent with 5-FU. There was no difference in the median survival (15.5 months in the chemoradiotherapy arm and 16.1 months in the no-chemoradiation arm). In the final results of the ESPAC-1 trial, the median survival was 15.9 months in the chemoradiotherapy arm and 17.9 months in the group not assigned to receive chemoradiotherapy (*P* = 0.05) [14]. The estimated 5-year survival was 10% in the chemoradiotherapy arm compared with 20% in those who did not receive chemoradiotherapy (*P* = 0.05).

With both EORTC and the ESPAC-1 studies showing no survival benefit, the evidence to support continued use of adjuvant chemoradiotherapy in pancreatic cancer has been markedly reduced. This leads to increased interest in clinical trials using chemotherapy alone.

The ESPAC-1 trial also studied the benefit of a bolus of 5-FU administered intravenously. A total of 289 patients were randomized using the 2 × 2 factorial design and followed for 47 months [14]. The survival with chemotherapy was 20.1 months and without chemotherapy was 15.5. The survival benefit was evident not only with R0 but also with R1 resection.

In contrast to contradictory data for chemoradiation therapy, clinical research with gemcitabine has shown it to be a major advance in the treatment of pancreatic cancer. Gemcitabine is a difluorinated analog of the naturally occurring nucleoside deoxycytidine and has shown significant clinical activity in a variety of solid tumors including pancreatic cancer. A most recent and significant study regarding the use of adjuvant gemcitabine is the CONKO-001 (Charité Onkologie) study [15]. This multicenter randomized control trial conducted between July 1998 and December 2004 was designed to test the hypothesis that adjuvant chemotherapy with gemcitabine administered after complete resection of pancreatic cancer improves disease-free survival by 6 months or more. A total of 368 patients with gross complete (R0 or R1) resection of pancreatic cancer and no prior radiation or chemotherapy were enrolled into 2 groups. One group of patients was randomized to receive adjuvant chemotherapy with 6 cycles of gemcitabine on days 1, 8, and 15 every 4 weeks (*n* = 179), and the second group was observed (*n* = 175). Median disease-free survival was 13.4 months in the gemcitabine group and 6.9 months in the control group. Estimated disease-free survival at 3 and 5 years was 23.5% and 16.5% in the gemcitabine group, and 7.5% and 5.5% in the control group, respectively. These authors concluded that treatment with gemcitabine for 6 months after complete resection of pancreas cancer statistically significantly increases median and disease-free survival. A recent abstract reporting followup in 2008 confirms these benefits [16].

The effect of gemcitabine on disease-free survival was significant in patients with either R0 or R1 resection. In the follow-up analysis gemcitabine did improve the overall survival (gemcitabine 22.8 months versus control 20.2 months). The most impressive statistic was the delayed development of recurrent disease after complete resection of pancreatic cancer compared with observation alone. This clinical trial strongly supports use of gemcitabine as adjuvant chemotherapy in resectable carcinoma of the pancreas.

Given the conflicting data concerning the use of chemoradiotherapy in resected pancreatic cancer, the optimal treatment of patients in this setting remains controversial. In Europe, chemotherapy with gemcitabine alone is generally accepted as standard of care, whereas in the United States, chemoradiation therapy is still commonly recommended.

Recently, a multiagent chemotherapy regimen used to treat patients with unresectable disease has shown increased survival when compared to single-agent Gemzar. In 342 randomized patients the FOLFIRINOX regimen resulted in a median overall survival of 11.1 months as compared to 6.8 months in the gemcitabine group. Clearly, this multiagent chemotherapy regimen becomes a candidate for adjuvant treatment of resected pancreas cancer [17].

## 4. Intraperitoneal Gemcitabine Pharmacokinetics

Gemcitabine is a prodrug which has little or no cytotoxic effect. The drug is metabolized within tissue to the active agent, gemcitabine triphosphate. The efficacy of gemcitabine has been correlated with concentrations of gemcitabine triphosphate accumulated in peripheral blood mononuclear cell (PBMC), which in turn is related to plasma concentration. The rate of intracellular accumulation of gemcitabine triphosphate was highest when plasma gemcitabine was about 20 micromol/L [18]. Beyond this there is enzymatic saturation, and further increase in plasma concentration does not produce any increase in intracellular gemcitabine triphosphate concentration.

There are two types of infusion regimens followed for gemcitabine. First is the fixed dose rate regimen: In this regimen generally 1,000 or 1,500 mg/m^2^ is infused during 100 or 150 minutes. The dose rate of 10 mg/m^2^/min achieves the target plasma concentration of 20 micromol/L.

In contrast the standard dose therapy of gemcitabine administered by intravenous infusion is 1000 mg/m^2^  over 30 minutes once weekly for up to 7 weeks (or until toxicity necessitates reducing or holding a dose), followed by a week of rest from treatment. Subsequent cycles should consist of infusions once weekly for 3 consecutive weeks out of every 4 weeks.

Much of the controversy about the use of gemcitabine in further clinical trials has concerned the possible superiority of fixed dose rate over the standard dose schedule. It is a known fact that the fixed dose rate infusion achieves better concentrations of gemcitabine triphosphate in PBMCs, but the clinical benefit of this is uncertain [18].

A criticism of the use of intraperitoneal gemcitabine in carcinoma of the ovary was that better plasma concentrations could be achieved by fixed dose rate infusion of gemcitabine than by intraperitoneal administration. In the study by Sabbatini et al. plasma concentrations of intraperitoneal gemcitabine administered were between 0.92–8.2 micromol which was considerably below the threshold for maximum effect (20 micromol) [19]. However, this criticism ignores the high likelihood that intraperitoneal chemotherapy acts by direct uptake of the drug into cancer cells or peritoneal implants. Furthermore, as Gandhi have pointed out, almost all pharmacokinetic studies on gemcitabine have a caveat that the cellular pharmacokinetic data are obtained from a surrogate tissue (circulating peripheral blood mononuclear cells) rather than from the target solid tumor tissue [18]. The gemcitabine drug levels within solid tumor tissue are not known. Also, levels of gemcitabine-activating and -inactivating enzymes within cancerous tissue such as cytidine deaminase, deoxycytidine kinase, and nucleotidases are not well defined. It is merely an assumption that fixed dose rate infusion in comparison to intraperitoneal administration would result in greater area under the curves (AUC) and/or peak levels of gemcitabine triphosphate in tumor cells located at the peritoneal surface of the abdomen and pelvis. Gandhi has suggested pharmacologic studies in which tumor tissue is directly available for measurement of gemcitabine triphosphate concentration.

Clinical and laboratory studies do show a theoretical advantage of intraperitoneal versus intravenous gemcitabine [20]. Pestieau and colleagues studied the pharmacokinetics of intraperitoneal gemcitabine in a rat model. The area under the curve ratio of intraperitoneal to systemic drug exposure in the rat model was between 12.5 and 26.8 depending on the dose of intraperitoneal gemcitabine. All tissue samples showed an increased drug concentration when administered with intraperitoneal hyperthermia as compared to a normothermic state.

Sugarbaker and colleagues reviewed the data on intraperitoneal gemcitabine in humans by taking plasma and peritoneal fluid samples from patients in the operating room [21]. These data showed that gemcitabine used with heated intraoperative intraperitoneal administration at 1,000 mg/m^2^ in 3 Liters had marked local-regional drug exposure. The area under the curve ratio of concentration times time for intraperitoneal to intravenous drug was 500. In this study of a patient who had resected pancreas cancer treated with intraperitoneal hyperthermic gemcitabine, considerable benefit was suggested by the pharmacologic data.

The adequate plasma concentration of 5.26 mcg/mL has been recommended [19]. In our patient presented in Figure 2, the peak plasma concentration was 4.03 mcg/mL, very close to the target achieved by a fixed-dose-rate infusion. Of course, the translation of the pharmacologic advantage into an improvement in local-regional disease control requires further clinical studies.

In a study involving nine patients with advanced pancreatic malignancy reported by Gamblin et al., intraperitoneal chemotherapy was administered using indwelling peritoneal catheters [22]. Intraperitoneal gemcitabine was well tolerated, and no significant toxicities were noted. There was rapid decrease in peritoneal gemcitabine concentration due to almost total absorption of the intraperitoneally administered gemcitabine. Steady plasma concentrations were reached early implying absorption of virtually all intraperitoneally administered gemcitabine. These findings combined with the fact that gemcitabine has low local toxicity argue well for its use in intraperitoneal chemotherapy.

## 5. Intraperitoneal Gemcitabine in Ovarian Carcinoma

A phase 2 study using intraperitoneal cisplatin and gemcitabine in carcinoma of the ovary was conducted by Sabbatini et al. [19]. The patients selected were those with persistent disease documented by a second-look assessment. The patients were given intraperitoneal cisplatin (75 mg/m^2^) on day 1 and intraperitoneal gemcitabine at 500 mg/m^2^ on days 1, 8, and 15 on a 28-day schedule for four courses. The median time to treatment failure and overall survival of 15.9 and 43.5 months, respectively, were consistent with historical data in second-look-positive patients receiving a variety of intraperitoneal platinum-based regimens for consolidation. There was no apparent benefit with intraperitoneal gemcitabine, and the authors attributed this to the dense peritoneal fibrosis that they encountered during second-look surgery. The authors of this study (as discussed earlier) have stated that the concentrations of intraperitoneal gemcitabine in peripheral blood mononuclear cells were determined to be much below the maximum therapeutic values in plasma. Data regarding an increased local-regional drug concentration and improved local-regional control of cancer as a result of intraperitoneal administration was not provided.

In the study by Sabbatini et al., patients were treated using intraperitoneal cisplatin at 75 mg/m^2^ on day 1 with a dose escalation of gemcitabine at 500, 750, 1000, or 1250 mg/m^2^ intraperitoneally on days 1, 8, and 15 of a 28-day schedule for four courses [19]. The phase I dose-limiting toxicity was grade 3 thrombocytopenia at day 15 on dose level 1. The chemotherapy protocol was modified to cisplatin (75 mg/m^2^) on day 1 and gemcitabine at 500 mg/m^2^ on days 1 and 8 of a 21-day schedule for four courses.

Of the 30 patients that were enrolled for the study, 9 were removed from the study; one each for hypersensitivity, cellulitis, and intraperitoneal port malfunction, two for progression of disease, and four for renal toxicity. Other toxicities included grade 3 nausea (7%) and transient grade 3 neuropathy (3%). Grade 1 or 2 neuropathy was frequently seen (80%). Five patients (17%) returned to the operating room at a median of 6 months (range, 1–20 months) after intraperitoneal therapy for evaluation of abdominal pain; two patients had recurrence, and all had areas of fibrous tissue with encasement of the bowel. The peritoneal sclerosis was, most likely, related to repeated doses of intraperitoneal cisplatin. The lack of benefit from intraperitoneal gemcitabine in ovarian cancer patients may be from poor drug distribution and extensive peritoneal fibrosis in this group of patients.

## 6. Clinical Trials of Gemcitabine Alone or in Combination with Other Drugs in Patients with Unresectable Pancreas Cancer

The current available evidence for treatment for pancreatic cancer suggests that gemcitabine monotherapy chemotherapy should be considered a valid treatment option. In the important study reported by Burris and colleagues, 126 chemotherapy-naïve patients with unresectable pancreatic cancer were randomized to receive either intravenous gemcitabine or 5-fluorouracil. The primary endpoint was a composite of pain measurements, weight, and performance status [23]. Patients treated with gemcitabine derived significantly more clinical benefit than those receiving 5-fluorouracil (23.8% versus 4.8%, respectively; *P* = 0.0022). In addition there was a statistically significant improvement in overall survival (median: 5.65 versus 4.41 months, resp.) with a 1-year survival rate of 18% in the gemcitabine cohort compared with 2% in patients receiving 5-fluorouracil (*P* < 0.002).

Berlin and colleagues published an ECOG phase 3 trial including 327 patients with advanced carcinoma of the pancreas [24]. They showed that 5-fluorouracil, administered in conjunction with gemcitabine, did not improve the median survival of patients with advanced pancreatic carcinoma compared with single-agent gemcitabine. The authors concluded that further studies with other combinations of gemcitabine and 5-fluorouracil are not compelling and clinical trial resources should address other combinations and novel agents. Several other chemotherapy agents have been tried in combination with gemcitabine.

Irinotecan with gemcitabine has not shown any benefit as compared to gemcitabine alone [25].

The combination of gemcitabine with cisplatin and oxaliplatin has been more encouraging. In a German multicenter study, Heinemann et al. enrolled 195 patients to receive either gemcitabine alone or in combination with cisplatin [26]. These results supported the efficacy and safety of an every-2-week treatment with gemcitabine plus cisplatin. Median overall survival and progression-free survival were more favorable in the combination arm as compared with gemcitabine alone, although the difference did not attain statistical significance. The French Multidisciplinary Clinical Research Group (GERCOR)/Italian Group for the Study of Gastrointestinal Tract Cancer (GISCAD) intergroup study compared gemcitabine plus oxaliplatin to gemcitabine alone [27]. The pooled analysis of the GERCOR/GISCAD intergroup study and the German multicenter study indicates that the combination of gemcitabine with a platinum analog such as oxaliplatin or cisplatin significantly improves progression-free survival and overall survival as compared to single-agent gemcitabine in advanced pancreatic cancer especially in patients with good performance status [28].

Scheithauer et al. reported on gemcitabine in combination with capecitabine [29]. A somewhat superior clinical benefit response rate was seen with the drug combination. However, no advantage over single-agent gemcitabine was noted in terms of objective efficacy parameters.

The combination of gemcitabine and mitomycin C was studied by Tuinmann et al. in a trial involving 55 patients with advanced pancreatic cancer [30]. These patients were given gemcitabine 800 mg/m^2^ intravenously on days 1, 8, and 15, and mitomycin C 8 mg/m^2^ intravenously on day 1 every 4 weeks in an outpatient setting. A median of 3 cycles was administered. The most frequent toxicity was thrombocytopenia grade III/IV seen in 54% of patients. The objective response rate was 29%. Eighteen patients had stable disease resulting in an overall tumor growth control of 62%. Time to progression was 4.7 months, and median overall survival was 7.25 months. The authors concluded that the combination was well tolerated. Survival was similar to monotherapy with gemcitabine.
